# Gender-Mediated Differences in Vertical Transmission of a Nucleopolyhedrovirus

**DOI:** 10.1371/journal.pone.0070932

**Published:** 2013-08-05

**Authors:** Cristina Virto, Carlos A. Zárate, Miguel López-Ferber, Rosa Murillo, Primitivo Caballero, Trevor Williams

**Affiliations:** 1 Instituto de Agrobiotecnología, CSIC-Gobierno de Navarra, Mutilva Baja, Navarra, Spain; 2 École des Mines d'Alès, Alès, France; 3 Departamento de Producción Agraria, Universidad Pública de Navarra, Pamplona, Navarra, Spain; 4 Instituto de Ecología AC, Xalapa, Veracruz, Mexico; Thomas Jefferson University, United States of America

## Abstract

With the development of sensitive molecular techniques for detection of low levels of asymptomatic pathogens, it becoming clear that vertical transmission is a common feature of some insect pathogenic viruses, and likely to be essential to virus survival when opportunities for horizontal transmission are unfavorable. Vertical transmission of *Spodoptera exigua* multiple nucleopolyhedrovirus (SeMNPV) is common in natural populations of *S. exigua*. To assess whether gender affected transgenerational virus transmission, four mating group treatments were performed using healthy and sublethally infected insects: i) healthy males (H♂)×healthy females (H♀); ii) infected males (I♂)×healthy females (H♀); iii) healthy males (H♂)×infected females (I♀) and iv) infected males (I♂)×infected females (I♀). Experimental adults and their offspring were analyzed by qPCR to determine the prevalence of infection. Both males and females were able to transmit the infection to the next generation, although female-mediated transmission resulted in a higher prevalence of infected offspring. Male-mediated venereal transmission was half as efficient as maternally-mediated transmission. Egg surface decontamination studies indicated that the main route of transmission is likely transovarial rather than transovum. Both male and female offspring were infected by their parents in similar proportions. Incorporating vertically-transmitted genotypes into virus-based insecticides could provide moderate levels of transgenerational pest control, thereby extending the periods between bioinsecticide applications.

## Introduction

Nucleopolyhedroviruses (genus *Alphabaculovirus*, family Baculoviridae) are arthropod-specific viruses that have been used in many parts of the world as biological insecticides due to their insecticidal properties towards certain insect pests and their outstanding biosafety characteristics [1]. They are also commonly employed in biotechnological applications for the production of recombinant proteins [2].

Nucleopolyhedrovirus populations adopt one of two transmission pathways to infect susceptible host insects. Horizontal transmission occurs when virus occlusion bodies (OB) from an infected cadaver are consumed in sufficient quantity by a healthy conspecific larva. This is, by far, the best understood mechanism of transmission [3]. Little is known about vertical transmission of entomopathogenic viruses from infected parents to their offspring, but this has been proposed as a survival strategy to overcome periods of host scarcity when opportunities for horizontal transmission are limited [3]. Clearly the ability to adopt horizontal or vertical transmission routes depends on the virulence of the infection; only persistent sublethal infections with reduced virulence will be capable of vertical transmission [4]. Persistent infections have been reported in a number of lepidopteran species from field-collected [5], [6], and laboratory populations [7], [8], [9], [10].

The beet armyworm, *Spodoptera exigua* is a major pest of greenhouse crops in many parts of the world [11]. The multiple nucleopolyhedrovirus of *S. exigua* (SeMNPV) has been developed as the basis for several bioinsecticide products [12]. Vertical transmission of SeMNPV was a common feature in field-collected adults of *S. exigua* in southern Spain [13]. In that study, a selection of vertically transmitted (VT) genotypes, were isolated and their insecticidal properties were characterized. Among these, the VT-SeAl1 genotype had the greatest capacity to induce persistent infections compared to genotypes associated with the horizontal transmission pathway. Sublethal infections by VT genotypes persisted for at least five generations after their inoculation in a healthy experimental laboratory colony of *S. exigua* [14]. Transgenerational transmission can involve the transovarial or transovum pathways. Transovarial transmission describes the process of virus passing to progeny within the eggs, whereas the transovum route involves contamination of the egg surface with viral particles that infect neonate larvae when they ingest the chorion [7], [15].

Unexpectedly, Cabodevilla et al. [13] observed that a fraction of field-caught gravid females produced virus-infected offspring even though no evidence of infection was seen in these females using sensitive PCR methods targeted at the detection of viral transcripts. This led us to suspect that these females may have mated with infected wild males, suggesting that both sexes could contribute to vertical transmission of the pathogen. In the present study we determined the effect of parental gender and the importance of the transovum *vs.* transovarial routes on the transmission efficiency of this virus.

## Materials and Methods

### Insects and viruses

A healthy *S. exigua* culture was obtained from Andermatt Biocontrol AG (Grossdietwil, Switzerland) and reared on artificial diet [16] at a constant temperature (25±1°C), relative humidity (50±5%), and photoperiod (16 h∶8 h light∶dark cycle) in the insectary facilities of the Universidad Pública de Navarra, Pamplona, Spain. A single genotype of SeMNPV, named VT-SeAl1, was used in the experiment. This genotype was previously isolated from a sublethally infected colony of insects collected in the greenhouses of Almeria (Spain) and was known to be capable of parent to offspring transmission [13], [14].

### DNA extraction

Total insect DNA was extracted using MasterPure Complete DNA Purification kit (Epicentre Biotechnologies) standard protocol for tissue samples. Abdomens of recently thawed adults were dissected individually, and sexed by observation of external genitalia. The dissected abdomen was placed in a 2 ml tube with ceramic beads and 300 µl of lysis solution with 1 µl of 50 µg/µl Proteinase K added. The tissue was homogenized using MP FastPrep-24 tissue in a cell homogenizer at 4.0 m/s for 20 s. The mixture was incubated at 65°C for 15 min with a constant 1100 rpm orbital agitation. A 150 µl volume of the sample was then treated with RNase for 30 min at 37°C. Debris was pelleted by adding protein precipitation reagent and centrifuged at 10000×g for 15 min. DNA was precipitated using cold isopropanol, washed twice with 70% ethanol, resuspended in 20 µl Milli-Q water and stored at −20°C. Blank extraction samples containing only water were processed in parallel to detect cross-contamination during the extraction process.

### Detection of sublethal infections

Quantitative PCR based on SYBR fluorescence was performed in an ABI PRISM 7900HT Sequence Detection System (Applied Biosystems) in 96-well reaction plates. To detect virus genomic DNA specific primers were designed to amplify a 149-bp region of the SeMNPV *DNA polymerase* gene (DNApol149-Fw: 5′-CCGCTCGCCAACTACATTAC-3′; DNApol149-Rv: 5′-GAATCCGTGTCGCCGTATATC-3′) based on the complete genome sequence of the SeMNPV strain VT-SeAl1 (unpublished data). Amplifications were performed in a total reaction of 10 µl containing 5 µl of SYBR Premix Ex Taq (2×), 0.2 µl of ROX Reference Dye (50×), 0.2 µl of both forward and reverse primers (10 pmol/µl), and 1 µl of DNA template containing up to 50 ng of DNA. Three non-template reactions were included in each run and a standard curve was prepared in duplicate to determine the efficiency of each reaction. The qPCR protocol consisted of an initial denaturation step at 95°C for 30 s, followed by 45 amplification cycles of 95°C for 5 s, 60°C for 30 s, and finally added dissociation steps of 95°C for 15 s, 60°C for 15 s, 95°C for 15 s. Data acquisition and analysis were handled by Sequence Detector System version 2.2.2 software (Applied Biosystems). For the standard curve VT-SeAl1 DNA was extracted from OBs, purified thorough CsCl gradients, quantified using a spectrophotometer (Eppendorf BioPhotometer plus) and then serially diluted in sterile MilliQ water to the following concentrations: 10, 1, 0.5, 0.1, 0.05, 0.01, 0.005, and 0.001 pg/µl. A total of seven replicates of the DNA dilutions were performed and the average Cq value for each point was calculated and used to fit a linear regression. DNA quantities were consistently estimated per sample by extrapolation of Cq values from the standard curve. Every DNA sample was performed in triplicate and the specificities of PCR products were monitored by analyzing amplification profiles and the corresponding dissociation curves. Quantified viral DNA was normalized based on the total DNA concentration for each sample and measured using a NanoDrop 2000 (Thermo Scientific).

### Gender effects on vertical transmission

To determine gender effects on vertical transmission of SeMNPV, groups of adults that were either sublethally infected (infected males: I♂ and infected females: I♀) or were not subjected to prior virus treatment (virus-free adults, healthy males: H♂ and healthy females: H♀). For this, two genetically identical subpopulations were generated by inducing sublethal infections in experimental insects (qPCR detection limits 10^−3^ pg of viral DNA), whereas insects from the healthy treatment groups were not subjected to virus inoculation. To produce sublethally infected insects batches of 200 newly molted *S. exigua* fourth instars were fed a virus suspension containing 9×10^3^ OB/ml. In parallel, groups of 100 larvae were treated identically using a suspension without OBs. Larvae that drank the suspension within 10 minutes were individually placed in perforated 25-ml plastic cups containing artificial diet and reared at 25±2°C and 50%±5% RH until pupation or death from virus disease. Pupae that survived inoculation were assigned to separate groups according to their sex and viral treatment. Once the adults emerged, the following mating schedules were performed: i) healthy males (H♂)×healthy females (H♀); ii) infected males (I♂)×healthy females (H♀); iii) healthy males (H♂)×infected females (I♀) and iv) infected males (I♂)×infected females (I♀). Five adult pairs were confined in groups in paper bags provided with a moist cotton water source and maintained at 25±2°C and 50±5% RH for oviposition during a 2–4 day period. Egg batches from each treatment group were collected using sterilized instruments and adults were frozen at −80°C for subsequent analysis (F_0_ generation). Egg masses laid from each paper bag were divided into two parts and either soaked in a 0.25 ppm sodium hypochlorite solution (surface decontamination) or in sterile distilled water (no decontamination) for 5 minutes. Groups of 24 larvae that emerged from each half of each egg mass were individually placed in 25 ml cups with diet, and reared individually through to the adult stage (F_1_) to avoid cross-contamination among insects in this cohort. Adults were individually stored at −80°C for subsequent analysis. The whole experiment was performed four times.

### Statistical analysis

In order to determine the influence of parental infection status on the offspring, the prevalence of qPCR positive insects and results of virus loads in the offspring were analyzed by fitting generalized models in GLIM 4 (Numerical Algorithms Group, Oxford, UK) with a binomial or normal error distribution specified, respectively, and with gender or mating group treatment specified as factors. Proportions of infected insects were compared by Fisher's exact test and subjected to *t*-test for pairwise comparison. Values of viral load per infected insect were normalized by log-transformation prior to analysis. The effect of egg surface decontamination on vertical transmission was examined using Pearson's χ^2^ test in the SPSS Statistics program (v.19 IBM). The correlation between proportions of F_1_ infected insects and their viral load was examined by Spearman's rank correlation.

## Results

### Establishing qPCR amplification parameters

Following mating and oviposition, parental insects from each of the four mating groups were subjected to qPCR to determine the prevalence of sublethal infection. A linear relationship was established between the critical quantification cycle (Cq) and the log-transformed amount of viral DNA (Figure S1). The regression coefficient (R^2^ = 0.996) and slope value (−3.215), indicated very high reaction efficiency [17]. The cut-off value was defined as the lowest concentration detected that fell within the linearity of the regression, in this case 1×10^−3^ pg. This value was used to set a limit of 33.4 cycles; all samples with higher Cq values were treated as negative, whereas all samples with a lesser number of cycles and that showed a single peak at the expected melting temperature (83°C) in the dissociation curve, were considered as positive. As the genome of VT-SeAl1 was estimated to be 135696 bp (unpublished data) the theoretical detection limit equates to 6.8 genome copies per reaction.

### Detection of sublethal infections in parental (F_0_) insects

**Figure 1:**
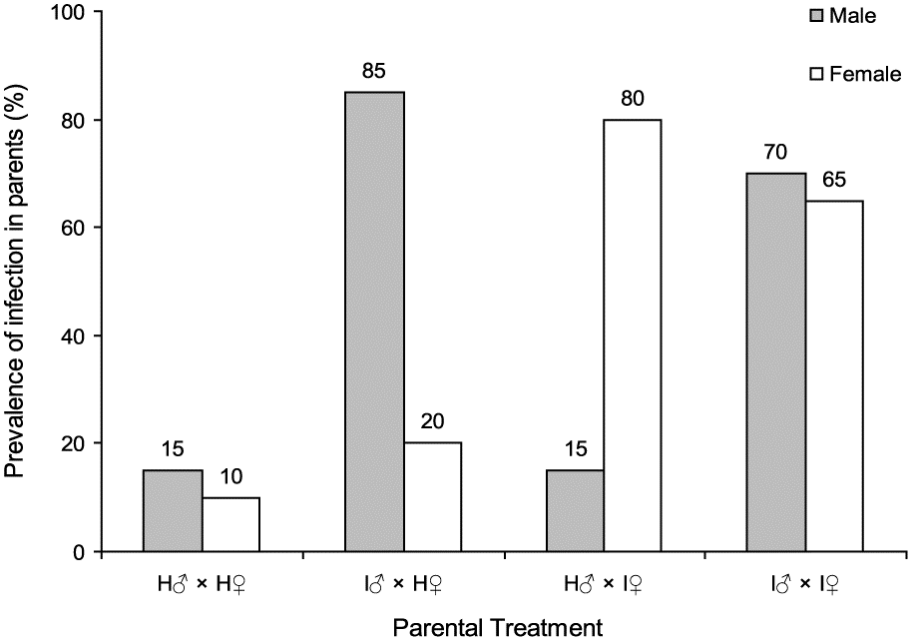
Prevalence of infection in parental adults in each mating group. H♂: Healthy male, H♀: Healthy female, I♂: Infected male, I♀: Infected female. Each mating group comprised 20 male and female *S. exigua* moths.

Overall, 57.6±4.4% of the larvae that consumed VT-SeAl1 OBs succumbed to virus infection, whereas no mortality was registered in mock-infected control larvae. The prevalence of qPCR positive reactions in the insects that survived, following consumption of viral OBs in the larval stage, was clearly higher than that of control insects (Figure 1). Viral load in parental (F_0_) adults averaged 1.514±0.287×10^−3^ pg viral DNA/µg total DNA per insect (N = 72 positive samples) that represents 10.34±1.96 genome copies per reaction.

Overall, sublethally infected individuals were more abundant in the virus challenged groups of insects than in the mock-infected groups (χ^2^ = 60.49, *df* = 1, *P*<0.001). Between 70 and 85% of adult males that survived OB treatment were classified as sublethally infected, compared with 65 to 80% of adult females (Figure 1). Unexpectedly, 12 out of the 80 insects that had not consumed OBs were positive for sublethal infection, suggestion a low level of inapparent or latent infection in the insect colony from Switzerland (Figure 1). The frequency of infected adults in the control group H♂×H♀ (5 positive adults (both sexes) out of 40 tested) was similar to that found in apparently healthy groups mated with infected insects (I♂×H♀ = 4 positive females out of 20 and H♂×I♀ = 3/20 positive males; χ^2^ = 0.392, *df* = 1, *P* = 0.531).

### Transgenerational transmission

**Figure 2:**
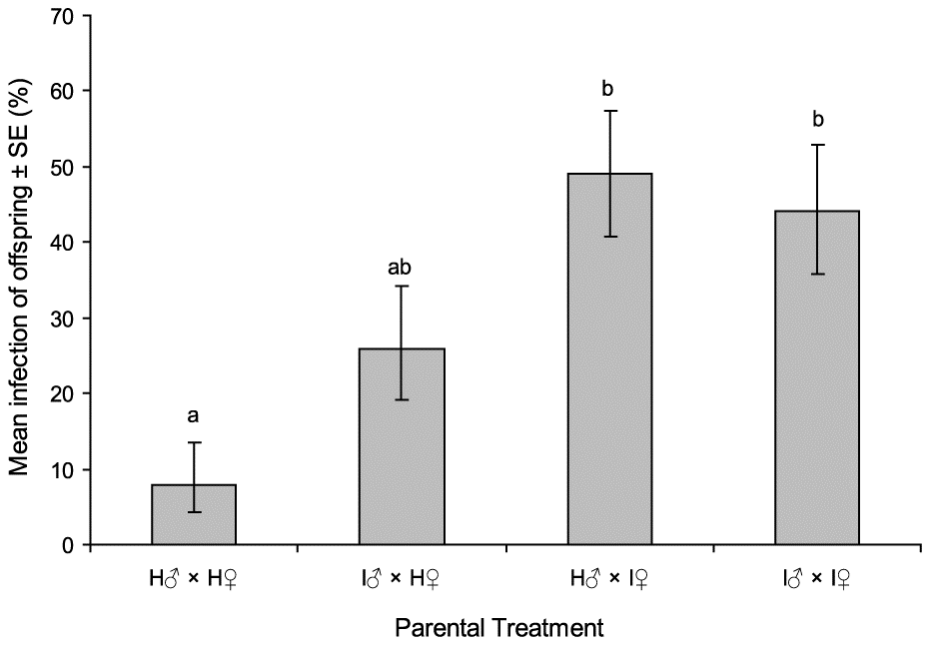
Percentage of offspring positive by qPCR according to parental infection status. H♂: Healthy male, H♀: Healthy female, I♂: Infected male, I♀: Infected female (N = 120). Columns labeled with different letters indicate significant differences (*t*-test, P<0.05).

In order to elucidate whether virus was passed to offspring via transovarial or transovum transmission, the prevalence of sublethal infection in F_1_ adults was compared between adults from either decontaminated or non-decontaminated eggs. Egg surface decontamination did not significantly affect the prevalence of sublethally infected F_1_ adults (decontaminated eggs = 16.5%; non-decontaminated eggs = 14.9%; χ^2^ = 0.649, *df* = 1, *P* = 0.420). Therefore, all results were pooled across decontamination treatments for subsequent analyses. Parental mating group treatment significantly influenced viral transmission to F_1_ adults (*F* = 18.95, *df* = 3, 31, *P*<0.001; Figure 2). Subletally infected males that mated with healthy females produced offspring with an average prevalence of 26% inapparent infection, compared to 8% in the offspring of the control insect group. In contrast, when infected females mated with healthy males, the prevalence of infection in offspring was 49%, compared to 44% when both parents were infected (Figure 2). These results indicate that female-mediated vertical transmission was approximately twice as efficient as male-mediated transmission. Both sexes of offspring were equally likely to have acquired a sublethal infection from their parents (male mean = 34.9±6.7%; female mean = 27.9±7.9%; *F* = 0.997, *df* = 1, 28, *P* = 0.327). Similarly, no significant interaction was observed between parental mating group and offspring gender in the prevalence of sublethal infection (F = 0.863, *df* = 3, 27, *P* = 0.472). Importantly, none of the F_1_ generation insects died of patent virus disease during rearing from larva to adult, so that all infections that we detected were present in insects that showed no signs of disease.

### Virus DNA loads present in offspring

Viral load in F_1_ adults was quantified and normalized by total DNA content for each insect sample. Mean viral load values in infected offspring were similar between mating groups (*F* = 1.31, *df* = 3, 12, *P* = 0.316) and ranged from 1.07±0.12×10^−3^ to 1.76±0.29×10^−3^ pg viral DNA/µg total DNA, i.e., the quantity of viral DNA in each insect was independent of the parental source of the infection (male, female or both).

In order to investigate whether viral loads differed according to offspring the viral load results of F_1_ infected adults were pooled and found not to differ significantly according to sex (male mean = 1.74±0.26 pg viral DNA/µg total DNA; female mean = 1.46±0.16 pg viral DNA/µg total DNA; *t* = 0.639, *df* = 25, *P* = 0.474).

**Figure 3:**
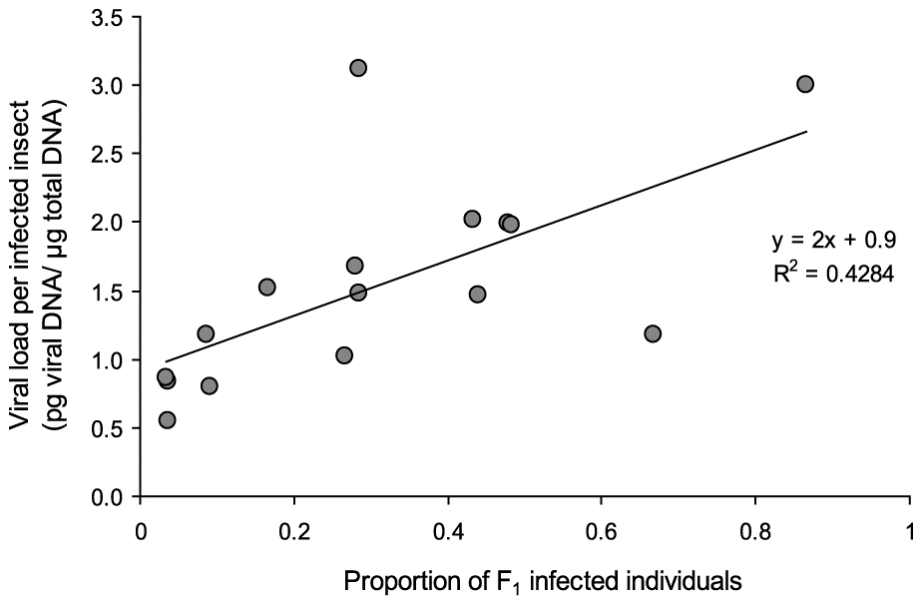
Relationship between viral DNA load per F_1_ infected insect and proportion of F_1_ infected insects in each cohort. Grey circles indicate different experimental groups taking into account parental treatment and replicate. (Spearman rank correlation: 0.687, P<0.05).

Finally, a significant positive relationship was detected between average viral load per F_1_ infected insect and the proportion of F_1_ infected insects produced by each mating group (Spearman rank correlation: 0.687, *P*<0.05), i.e., the prevalence of vertical transmission was positively associated with the number of genome copies in each infected insect (Figure 3).

## Discussion

The development of highly sensitive molecular tools has recently allowed insect pathologists to focus attention on the vertical transmission of insect viruses and to assess the role of this strategy in the survival of these pathogens in natural and laboratory insect populations [6]. It seems that both alphabaculoviruses [6], [18], [19], and betabaculoviruses [4] can establish sublethal infections in larvae that survive after having consumed OBs. Moreover the prevalence of such infections can be dose-dependent [14]. In the present study between 65 and 85% of sublethal infection was detected in adult survivors of an inoculum that killed 57.6% of experimental insects. A small number of control insects proved positive for sublethal infection by qPCR which suggests a low level infection in what was believed to be a completely healthy insect colony. However, it was clear that deliberately infected insects harbored markedly higher levels of virus than the untreated insects from the laboratory colony, which led us to believe that the overall findings and conclusions of this study are likely to be valid, despite the low level presence of virus in the host colony. Indeed, apparently healthy laboratory colonies of lepidopteran species are often found to harbor sublethal virus infections as soon as they are subjected to sensitive molecular techniques for pathogen detection [4], [6], [20], [21]. Moreover, latent infections have been reported for all developmental stages of asymptomatic individuals of *S. exigua*, confirming that inapparent infections can be detected in all stages of the host life cycle [10].

Our study demonstrated biparental transmission of SeMNPV to offspring, although the efficiency of maternally-mediated transmission was approximately double that of paternally-mediate transmission. Transmission during mating has been described in a range of insect pathogenic viruses, including a rhabdovirus in a palm beetle [22], a parvovirus in a mosquito [23], sigmaviruses of *Drosophila* spp. [24], an iflavirus in honeybees [25], and nucleopolyhedroviruses of lepidopteran pests [26], among others. Sexual transmission has also been demonstrated for the gonad specific nudivirus Hz-2V of the noctuid moth *Helicoverpa zea*, that was transmitted during copulation, through waxy virus-rich secretions at the tip of the abdomen of the infected insect [27].

Virus titers required for transmission were estimated to be very low and were calculated at approximately 10.4 viral genomes per reaction or 208 genomes per infected insect (assuming 100% efficiency in DNA extraction, which is highly unlikely). For the vertically-transmitted sigmavirus of *Drosophila* spp., the transmission of viral particles occurs inside the oocyte, likely due to the size and activity differences between male and female gametes. Infected male sperm may also be not as competitive as non-infected counterparts [28]. Accordingly, sigmaviruses show marked parental sex differences in the contribution to virus transmission and quantity of virus genomes transmitted to offspring [29], [30].

Persistent infections of nucleopolyhedroviruses often have biological costs that include lower developmental rates, lower pupal and adult body weights and reduced reproductive capacity [7], [14], [31], [32], [33], although occasionally beneficial effects have been detected [34].

The quantity of viral DNA present in sublethally infected insects of F_1_ did not differ significantly according to sex or mating group (infected fathers *vs.* infected mothers, or both), whereas lower titers of sigma virus were detected in *Drosophila* embryos when sigmavirus was paternally transmitted [30].

A positive correlation was detected between the percentage of infected adults in F_1_ generation and their viral load, suggesting that the adults that transmitted the virus to a high proportion of their progeny tended to transmit greater amounts of viral DNA. It may be that due to their genotype, nutritional or physiological characteristics, certain hosts provide better conditions for virus multiplication, so that they provide a greater contribution to the number of virus genome copies in the offspring. Further studies are required to investigate this hypothesis.

Previous studies on baculovirus transmission demonstrated that both sexes were involved in vertical transmission for *Bombyx mori* nucleopolyhedrovirus (BmNPV) [21], *Plodia interpunctella* granulovirus (PiGV) [4] and *Spodoptera exempta* nucleopolyhedrovirus (SpexNPV) [15]. Interestingly, viral particles were observed in either testis or ovaries cells, confirming the presence of the virus in gonads of sublethally infected individuals by histological observation [18] or by viral transcript detection [4], [18]. Vilaplana et al. [15] observed lethal NPV infection in offspring in both cases, when the infected parental was male or female. For *B. mori*, mating pairs with a female infected with BmNPV resulted in higher mortalities of first instar offspring (78%) than observed in offspring from treatments in which the male was responsible for transmission (57%). These authors concluded that transmission occurred principally via the transovarial route rather than transovum transmission [18], as did studies on SpexNPV on *S. exempta* in which the prevalence of infection in the offspring was independent of eggs surface decontamination treatment [15]. In contrast, in the present study patent disease was not observed in the offspring of covertly infected insects, perhaps as a result of low levels of stress during rearing or another unidentified factor that favored the maintenance of sublethal infection over the expression of patent lethal disease [7]–[9].

In conclusion, vertical transmission of SeMNPV was observed when male or female parents harbored a sublethal infection, but female-mediated transmission was more efficient than that of males. Improving our knowledge on the factors affecting vertical transmission mechanisms may contribute to the development of optimal strategies for the use of virus-based insecticides. Additional studies on the mechanisms that trigger sublethal infections into lethal patent infections may also provide useful information aimed at reducing the frequency of virus insecticide applications in the field.

## Supporting Information

Figure S1 **Standard curve for qPCR quantification.** Linear regression with different critical quantification PCR values (Cq) following serial dilution of *Spodoptera exigua* multiple nucleopolyhedrovirus (SeMNPV) genomic DNA.
(TIF)
